# Recovery of Visual Function in a Patient with an Onodi Cell Mucocele Compressive Optic Neuropathy Who Had a 5-Week Interval between Onset and Surgical Intervention: A Case Report

**DOI:** 10.1155/2010/483056

**Published:** 2010-10-12

**Authors:** Wencan Wu, Michelle T. Sun, Paul S. Cannon, Shi Jianbo, Dinesh Selva

**Affiliations:** ^1^Eye Hospital of Wenzhou Medical College, Wenzhou, Zhejiang 325027, China; ^2^Oculoplastic and Orbital Division, Discipline of Ophthalmology and Visual Sciences, The University of Adelaide and South Australian Institute of Ophthalmology, Adelaide, SA 5000, Australia; ^3^The Department of Otorhinolaryngology, First Affiliated Hospital of Sun Yat-sen University, Guangzhou 510080, China

## Abstract

*Purpose*. To report on a patient with compressive optic neuropathy secondary to an Onodi cell mucocele, who fully recovered visual function following surgery. *Method*. Case report. *Results*. A 28-year-old male was admitted with a right visual acuity of 20/100 following treatment for an initial diagnosis of optic neuritis. Subsequent examination suggested compressive optic neuropathy, and neuroimaging confirmed the presence of an Onodi mucocele compressing the optic nerve. The patient underwent a right endonasal sphenoethmoidectomy with decompression 5 weeks after the initial onset of symptoms. Three weeks following surgery, the visual acuity was 20/20, and there was complete resolution of the visual field defect, which has remained stable at 1 year. *Conclusion*. Onodi cell mucocele should be included in the differential diagnosis of a young patient with compressive optic neuropathy. Surgical decompression should be considered even when symptoms have been present for over a month.

## 1. Introduction

The Onodi cell is recognized as an anatomical variant, where the most posteriorly-positioned ethmoid cells enlarges into the body of the sphenoid bone. It has an identifiable optic canal bulge on endoscopic examination [1]. Onodi cell mucocele is an extremely unusual cause of compressive optic neuropathy. Its clinical significance relates to the relative position of the Onodi cell to the optic nerve. The pathogenesis is thought to be that of direct mechanical compression from the enlarging mucocele causing a subsequent circulatory disturbance with ischaemia resulting in an optic neuropathy [2, 3]. This paper is of optic neuropathy secondary to an Onodi cell mucocele in a young Chinese patient who had complete recovery of visual function following surgical decompression despite a relatively long time interval between the onset of symptoms and surgery. This is the first case, to our knowledge, of full recovery of visual function in a patient with optic nerve compression secondary to Onodi cell mucocele, where symptoms had been present for over a month prior to surgical intervention.

## 2. Case Report

A 28-year-old Chinese male was referred to a tertiary ophthalmic centre with a sudden decrease in right visual acuity (VA). Two weeks previously, the patient presented to a district hospital with a twenty-day history of progressive right visual loss associated with pain in his right eye and right-sided headache. The right best-corrected visual acuity was 20/50. The patient received intravenous ceftriaxone and methylprednisolone for presumed right optic neuritis. After five days of corticosteroid therapy, the vision in the right eye improved to 20/25 with resolution of the ocular pain and the headache. On the sixth day, the patient had a sudden deterioration in right visual acuity and was referred to an Orbital unit in a university teaching hospital for further management.

On admission to the tertiary centre, the right VA had deteriorated to 20/100. The patient had right ocular pain on eye movements. There was no previous ophthalmic, nasal, or medical history. On examination a right relative afferent pupil defect (RAPD) and a constricted right visual field on confrontation were recorded. The remainder of the ophthalmic and neurological examination was unremarkable. Fundoscopy revealed a pale right optic disc. A working diagnosis of compressive optic neuropathy was made, and further investigations were requested.

**Figure 1:**
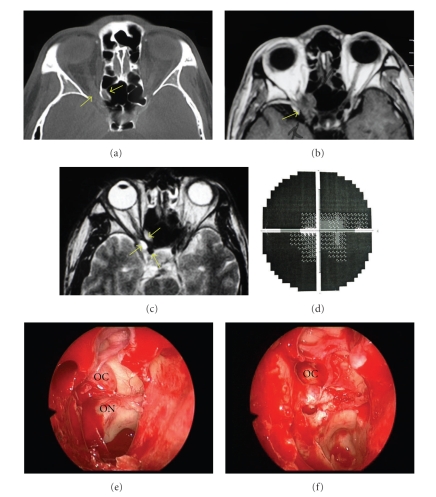
(a) Orbital CT imaging of a right Onodi cell mucocele compressing the optic nerve (arrows). (b) T1-weighted MRI image showing a hypointense signal due to the water content in the Onodi cell (arrows). (c) T2-weighted MRI image with a high signal intensity within the Onodi cell (arrows). (d) Humphrey visual field of the right eye with significant constriction of the visual field. (e) A clinical photograph of the Onodi cell mucocele (OC) and the optic nerve (ON) prior to decompression. (f) A clinical photograph following marsupialisation of the Onodi cell and drainage of the mucocele (OC).

Computed tomography (CT) scans revealed a dense homogenous mass in the posterior ethmoid cell, which was superior to the sphenoid sinus extending superolaterally around the right orbital apex (Figure 1(a)). Magnetic resonance imaging (MRI) confirmed the presence of an oval-shaped lesion compressing the right optic nerve (Figures 1(b) and 1(c)). A Humphrey visual field documented the marked tunnel vision in the patient's right eye (Figure 1(d)).

Thus, the neuroimaging confirmed that the compressive optic neuropathy was secondary to an Onodi cell mucocele. A right endonasal sphenoethmoidectomy with decompression of the Onodi mucocele was performed five weeks after initial presentation. The mucocele was drained of purulent fluid and marsupialised. The mucosa of the Onodi cell was found to be thickened and oedematous. The optic nerve was found to be exposed within the mucocele with dehiscence of the optic canal. The patient was commenced on a three-day course of 500 mg intravenous methylprednisolone and a five-day course of ceftriaxone.

Five days following surgery, the patient's RVA was 20/25 and improved to 20/20 three weeks later. The visual field defect had completely resolved. The patient has remained stable at one year.

## 3. Discussion

The incidence of Onodi cells varies from 8% to 13% on radiological findings [2] but has a much higher incidence (60%) on anatomic dissection [4]. Neuroimaging is central to the detection of the lesion, and coronal and sagittal views are recommended, as axial CT images alone may not reveal the mucocele if the thickness of the slices used is greater than the width of the Onodi cell [5].

A review of the current literature confirms that the mucocele is more common in Asia, with ten cases (91%) observed there. The mean age of the patients was 55 years (range 41–79 years), which is considerably older than the age of our patient.

Ten patients underwent an endoscopic transnasal approach to decompress the Onodi cell and one patient underwent a pterional craniotomy. Seven patients had good visual acuity recovery following surgery [3, 4, 6–9]. Of these patients, the time interval between presentation and surgery ranged from a few days [2] to three weeks [9]. Presenting visual acuities ranged from 20/50 [3] to hand movements [6], and postoperative acuity improvement ranged from 20/30 [7] to 20/20 [4, 6–8]. Six patients received corticosteroid therapy either prior to or following the surgical decompression. Three patients had previous sinonasal conditions [3, 7, 10], which may have increased the possibility of blockage of the ostium and formation of the mucocele. Our patient had no previous nasal pathology.

Surgical decompression should be attempted in all patients. Nonaka et al. [2] postulate that prognosis is related to the time interval between onset of symptoms and surgery, stating one month as a cutoff for poorer outcome. In the literature two patients failed to recover visual acuity, when surgery was undertaken more than two weeks after onset of symptoms. One patient had symptoms for fourteen days with no response to corticosteroids. No optic nerve exposure was observed during surgery [2]. The second patient had a five-month history of symptoms and was found to have an oedematous optic nerve during the surgery [10]. Our patient had symptoms for a month prior to decompression and was found to have an exposed optic nerve with resorption of the optic canal bone, both features are perceived to be poor prognostic factors in the recovery of visual function.

Visual acuity can improve on surgical decompression of the mucocele undertaken over a month after the onset of symptoms, as shown in our patient. We believe that surgery should be considered in all patients presenting with optic neuropathy secondary to an Onodi cell mucocele.
